# 

**Published:** 1898-03

**Authors:** 


### INDEX TO VOLUME XV.

ORIGINAL COMMUNICATIONS—

Abortion ........ 803
Accessory Sinuses of the Nose, Empyema of ........ 145
Ankle, Gunshot Wound of ........ 577
Ano, Fistula in ........ 23
Appendiciti.s, Diagnosis of ........ 1
Aseptic Surgical Technique, Possibilities of ........ 649
Asthma, Due to Intranasal Pressure ........ 669
Biliary Concretions, Treatment of ........ 520
Breast, Amputation of ........ 577
Cancer, Treatment of ........ 592
Cases, Abdominal ........ 444
Cases from Note-book ........ 235
Children, Mentally Deficient ........ 155
Chloroform Anesthesia, Danger of ........ 526
Cleft Palate ........ 367
Consumption ........ 228
Digit, Supernumerary ........ 577
Drug Eruptions, Cases of ........ 90
Drug Habit, Cases of ........ 80
Fractures, Treatment of ........ 742
Gonorrhea, Recurrent ........ 433
Gude's Pepto-Mangan, Remarks on ........ 93
Hermaphrodism, A Case of ........ 174
Infant Feeding ........ 373
Intestinal Obstruction, Technics of the Operation ........ 721
Iridectomy, Value of in Opaque Cornea ........ 585
Larynx, Syphilitic Manifestations in ........ 223
Mastoid, Inflammation of ........ 164
Medical Examining Board of Georgia ........ 589
Mouth-breathing in Children from Adenoids ........ 217
Obstetrical Practice, Some Complications in ........ 673
Orbit, Tumor of ........ 577
Otomyasthenia ........ 18
Pain, Pathology of ........ 73
Pregnancy, Diagnosis of ........ 798
Quarantine, National *vs.* State ........ 10
Reminiscences, Medical ........ 14, 86, 293, 582
Retinal Surgery in Strabismus ........ 807
Scott's Emulsion in Eye Trouble ........ 451
Shoulder, Pathology of Triple Articulation ........ 511

ORIGINAL COMMUNICATIONS—*continued*—

Suprapubic Cystotomy for Stone .... 793
Surgical Cases, Report of .... 522
Surgical Work, Some Recent .... 361
Third Tonsil, Hemorrhage After Removal of .... 170
Tonsillar, Abscess of .... 296
Tuberculosis of Bones .... 505
Turbinate Hypertrophies, Mental Disturbances in .... 306
Typhoid Fever, Serum Diagnosis of .... 440
Uterine Drainage .... 313
Uterus, Malignant Disease of .... 289
Vaccination and Its Sores .... 658
What Kind of Shoes We Should Wear .... 810

SOCIETY REPORTS—

Atlanta Society of Medicine .... 96, 238, 676, 815
Chicago Gynecological Society .... 603
Medical Association of Georgia .... 177
Quarantine Convention at Mobile .... 27
Richmond Academy of Medicine .... 528
Southern Surgical and Gynecological Association .... 725

CORRESPONDENCE—

Case, Report of .... 459, 530
Coca, Information Wanted .... 37
Examining Board of Georgia .... 613
Macon, Medical Items .... 531
Madan, Dr. D. Domingo .... 533
Medical Education, The Progress of .... 611
Needles, Hypodermic .... 110
Negro, The Color of When Born .... 384, 460, 530
New York Letter .... 105, 454
Osteopathy .... 530
Pan-American Medical Congress .... 679
Reeves, Dr., Letter from .... 743
Services, Compensation for .... 35
Spinal Curvature and Gymnastics .... 242
Vaccination, Painless .... 36
Warren, W. C .... 814

EDITORIALS—

Advertisements of Medical Journals .... 539
American Laryngological, Rhinological and Otological Society .. 45, 114
American Medical Press .... 468
Anesthetist a Specialty .... 540
Antitoxin of Park, Davis & Co .... 622
Antitoxin, Patent on .... 467
Atlanta College of Physicans and Surgeons .... 325
Atlanta Medical and Surgical Journal .... 756, 827

EDITORIALS—*continued*—

Atlanta Society of Medicine .......... 829
Atlanta Society of Medicine, Officers of .......... 756
Bourns, Dr. Frank S. .......... 755
Bubonic Plague in Vienna .......... 685
Cataract, Absorption of .......... 753, 754
Christian Science .......... 825
Christian Science and Death .......... 681
Cigarette, The Deadly .......... 247
Cocaine Ordinance .......... 691
Commencements, Medical College .......... 115
Consumption, The New Cure for .......... 385
Denver Meeting of A. M. A. .......... 322
Ear Disease and Life Insurance .......... 822
Exchange System, Stoppage of .......... 751
Georgia Medical Association .......... 111, 195, 620
Grandy, Dr. L. B. .......... 832
Gynecology, Conservative .......... 245
Hair-Dressing Parlors, Hygiene of .......... 749
Hamilton, Dr. Jno. B., Death of .......... 831
Hernia in Children .......... 545
Insanity, The Plea of .......... 43
Investigating Committee of War .......... 690
Kentucky, The, School of Medicine .......... 544
McRae, Dr. F. W. .......... 620
Medical Age, The .......... 621
Medical Colleges and A. M. A. .......... 465
Medical Colleges, Consolidation of .......... 389
Medical Examiners, State Board of .......... 475, 546
Medical Fortnightly, The .......... 544
North Carolina Medical Journal .......... 832
Olmsted Bill .......... 474
Ophthalmia Neonatorum, Law to Prevent .......... 41
Osteopathy .......... 535
Philadephia Polyclinic Journal .......... 831
Physicians' Accounts .......... 387
Physician, A, Does it Pay to Study? .......... 687
Physicians, Do They Know too Much? .......... 616
Plagiarism .......... 830
Pott's Disease .......... 543
Quarantine Convention at Atlanta .......... 193, 116
Quarantine Convention at Memphis .......... 745, 752
Quarantine Convention at Mobile .......... 39
Quarantine Question .......... 618
Queries, Medical .......... 542
Railway Surgeons, Southern Association of .......... 249
Smith, A. Lapthorn .......... 543
Southern Medical and Atlanta Medical Colleges .......... 251

EDITORIALS—*continued*—

Southern Surgical and Gynecological Association.................. 546
St. Paul Medical Journal.......................................... 832
Subscribers—Notice to.............................................. 475
Times and Register, The............................................ 542
Transplantation of Cornea.......................................... 828
Tri-State Medical Association...................................... 688
Tri-State Medical Journal.......................................... 830
University of Nashville............................................ 545

MEDICAL ITEMS..........49, 118, 197, 252, 327, 391, 479, 547, 623, 693, 757

BOOK REVIEWS—

*A Clinical Text-Book of Medical Diagnosis*............................ 699
*A Compend of Diseases of the Skin*.................................... 398
*A Country Doctor*..................................................... 488
*A History of Yellow Fever*............................................ 561
*A Manual of Modern Surgery*........................................... 488
*A Manual of Obstetrics*............................................... 58
*A Manual of Otology*.................................................. 663
*A Manual of Skin Diseases*............................................ 558
*A Manual of Venereal Diseases*........................................ 700
*A Primer of Psychology and Mental Disease*............................ 701
*A System of Medicine*................................................. 557
*A System of Practical Medicine*....................................... 56
*A System of Practical Medicine*....................................... 400
*A Text-Book of Materia Medica and Therapeutics*....................... 734
*A Text-Book of Mechano-Therapy*....................................... 843
*A Text-Book of Pathogenic Bacteria*................................... 560
*A Text-Book of Pathology*............................................. 767
*A Text-Book of Practical Therapeutics*................................ 632
*A Treatise on the Science and Practice of Medicine*................... 702
*Acromegaly* (*Hinsdale*).............................................. 842
*American Text-Book of Genito-Urinary Diseases*........................ 206
*American Year-Book of Medicine and Surgery*........................... 122
*An American Text-Book of Diseases of Children*........................ 559
*An American Text-Book of Gynecology*.................................. 561
*Annual and Analytical Cyclopedia of Medicine*......................... 203
*Atlas and Abstract of the Diseases of the Larynx*..................... 398
*Atlas and Epitome of Operative Surgery*............................... 488
*Atlas of Clinical Investigation*...................................... 204
*Atlas of Legal Medicine*.............................................. 335
*Atlas of Syphilis and Venereal Diseases*.............................. 490
*Brief Essays on Orthopedic Surgery*................................... 258
*Clinical Diagnosis*................................................... 58
*Commissioner of Education, Report of*................................. 398
*Compendium of Insanity*............................................... 206
*Conservative Gynecology*.............................................. 399

BOOK REVIEWS—*continued*—

*Compend of Obstetrics* .... 701
*Day-Dreams of a Doctor* .... 205
*Diseases of the Skin* .... 336
*Diseases of the Stomach* .... 54
*Diseases of the Stomach* .... 562
*Diseases of Women* .... 122, 563
*Glaucoma: Its Symptoms, Varieties, etc* .... 563
*Hay Fever and Its Successful Treatment* .... 400
*Histology, Elements of* .... 562
*Histology, Normal and Morbid* .... 770
*Human Anatomy* .... 766
*Hygiene of the Voice* .... 770
*International Medical Annual* .... 124
*King's American Dispensatory* .... 768
*Lectures on Appendicitis* .... 53
*Lectures on Tumors* .... 489
*Orthopedic Lectures* .... 841
*Pathology and Morbid Anatomy* .... 700
*Polk's Directory* .... 635
*Practical Diagnosis* .... 634
*Practical Urinalysis and Urinary Diagnosis* .... 767
*Prompt Aid to the Injured* .... 258
*Spinal Caries* .... 55
*Text-Book on Surgery* .... 57
*The Care and Feeding of Children* .... 123
*The Care of the Baby* .... 769
*The Medical News Pocket Formulary* .... 842
*The Office Treatment of Hemorrhoids* .... 490
*The Phonendoscope and its Practical Application* .... 839
*The Principles and Practice of Medicine (Osler)* .... 840
*The Sexual Instinct* .... 841
*The Surgical Complications of Typhoid Fever* .... 204
*The Treatment of Choleraic Diarrhea* .... 398
*The Year-Book of Treatment* .... 124
*Transactions of the American Orthopedic Association* .... 843
*Tuberculosis of Genito-Urinary Organs* .... 56
*Yellow Fever* .... 334

SELECTIONS AND ABSTRACTS—

Preservation of the Hymen, 706; Some Observations on Brain Anatomy, 709; Observations upon the Treatment of Some Cases of Neurasthenia, 711; Should We Drink at Our Meals? 638; The Sterno-mastoid Tumors of Children, 640; The Tongue as a Clinical Guide in Disease; 641; The Foramen of Winslow, 643; Removal of entire Stomach, 644; What a Trained Nurse Needs besides a Technical Training, 566; Should the General Practitioner Receive a Fee for Referring Cases to the Specialist? 670; Early Diagnosis of Tuberculosis of the Lungs, 572; Chronic

SELECTIONS AND ABSTRACTS—*continued*—

Ulceration of Cornea, 574; Velander's Pillow-slip Method of Mercurial Inunction, 574; Iodine in Obstinate Vomiting, 575; Treatment of Ivy Poisoning, 575; A Painless Blister, 576; The Relief of Fever in Tuberculosis, 576; Obedience, 576; Treatment of Locomotor Ataxia by Systematic Exercise, 493; The Bearing of Pathological Processes on Therapy of Morbid Conditions along the Genito-Urinary Tract in the Male, 501; Should all Milk Used for Infant Feeding be Heated for the Purpose of Killing Germs? 402; Professor Schenck's Researches on the Predetermination of Sex, 408; Inhalation of Vinegar to Control Nausea and Vomiting after Anesthesia, 411; The Compression Treatment of Pulmonary Tuberculosis, 413; Surgeon Nicholas Senn, 415; Normal Salt-solution, 417; The Management of Patients before and after Laparotomy, 419; Injection of Alcohol in Carcinoma, 420; The Non-Surgical Treatment of Pyelitis, 421; Serum Therapeutics and Prophylaxis of Yellow Fever, 422; The Physician as a Business Man, 423; Narcotic Notes, 424: The Hypodermic Injection of Quinine, 425; Care of Patients after the Operation for Appendicitis, 425; Amylolytic Ferments, 426; Malaria, 427; Prevention of Glaucoma, 427; Trichloracetic Acid, 428; Croupous Tonsillitus, 428; Record of Medico-Surgical Practice with Auxiliary Blood Supply, 429; Long, the Discoverer of Anesthesia, 338; Chronic Gastritis, 342; Diagnosis of Tumors of the Breast, 345; Aphthous Stomatitis, 349; Family History in Relation to Life Insurance, 354; Yellow Fever and the War, 356; Proceedings of the St. Louis Medical Society, 359; Modification of Cow's Milk, 260; Some Points in the Examination for Life Insurance, 264; Clean Surgery in Minor and Emergency Cases, 268; Modern Gunshot Wounds, 272; Curettage and Packing the Uterus, 274; Social and Professional Visits, 276; Christian Science and Science, 277; The Anti-Vivisection Craze, 278; A Decision of Importance to Physicians, 280; Malaria and the Cuban Campaign, 281; Results in 100 Abdominal Operations, 282; Scientific American Navy Supplement, 283; Fracture of the Neck of the Femur in Children, 284; Brain Abscess in Infants, 285; Anemia, 285; Diabetic Albuminuria and Its Treatment, 286; Woodbridge Treatment of Typhoid, 287; Care of the Sick and the Wounded, 287; How to Avoid Catching Consumption and How to Avoid Giving It to Others, 126; The Objects and Limits of Operation for Cancer, 130; Purulent Ophthalmia of the New-born, 133; Treatment of Gout, 135; Hydrozone and Glycozone in the Treatment of Gonorrhea, 137; The Tonsil and the Carotid Artery, 138: Should Cities Go into the Drug Business? 139; Morphine Anesthesia, 140; The Question of the Transformation of Calomel into Corrosive Sublimate in the Organism, 140; The Medical Excursion in June to Denver and Salt Lake City, 141; Injections of Alcohol in Carcinoma, 141; The Hand in Obstetrics, 141; How to Make a Mustard Plaster, 142; Delicate Test for Sugar, 142; Progress of Serum-Therapy, 60; The Treatment of Pulmonary Tuberculosis by the Hypodermic Use of a Compound Solution of Iodine, 70; Some "Don'ts" about Heart Disease, 71; Diffuse Scleroderma, 207; Mercurial Eruptions and Eruptions from Mercurial Inunc-

SELECTIONS AND ABSTRACTS—*continued*—

tion, 207; A Case of Disseminated Carcinoma of the Trunk, 208; Arsenical Pigmentation, 208; Cancer of the Urethra, 209; Liquid Air, 209; Possibilities of Antitoxin in Diphtheria, 211: Silkworm Gut, 213; The Negro Problem, 214; Cancer of the Breast, 215; "Knock-out Drops," 215; Bill Nye and the Nurses, 772; Nerve Fatigue of School Children, 775; The Use of Midwifery Forceps, 778; Ambulance Classes—A Beautiful Fad, 782; Physicians' Accounts and the Law, 783; The Urine as a Diagnostic Factor, 785; Uterine Cough, 786; Psoriasis, 787; Fasting in the Treatment of Infectious Diseases, 788; Extract of Supra-venal Capsule, 788; Relationship between the Medicinal Dose and the Peculiarities of the Growing Organism, 789; Quinine in Malarial Hemoglobinuria, 847; Treatment of Tetanus by Means of Intracerebral Injection of Antitoxin, 850; Pneumonia in Children, 852; Excision of Carbuncles, 855; Antistreptococcus Serum in Chancroid, 856; Faradaism in Dilatation of the Os Uteri, 857; Solomon Right Again, 858; Muscular Atrophies of Hysterical Origin, 858; Local Anesthesia, 859; A Tropical School of Medicine in London, 859; Uses and Effects of Manganiferous Iron Peptone, 860.

---

### LIST OF CONTRIBUTORS TO VOLUME XV.

---

Adams, W. E. .......... Greensboro, Ga.
Anthony, E. R. .......... Griffin, Ga.
Barnum, R. E. L .......... Richland, Ga.
Campbell, J. L. .......... Atlanta.
Carson, M. T. .......... Griffin, Ga.
Collins, Katherine .......... Atlanta.
Cooper, H. P. .......... Atlanta.
Corson, E. R. .......... Savannah, Ga.
Cox, R. P. .......... Rome, Ga.
Crichton, L. M. .......... Atlanta.
Crowe, W. A. .......... Atlanta.
Davis, E. C. .......... Atlanta.
Draper, T. J. .......... Mineral Springs, Ark.
Elkin, W. S. .......... Atlanta.
Frank, Louis .......... Louisville, Ky.
Galloway, W. C. .......... Wilmington, N. C.
Garlington, T. R. .......... Rome, Ga.
Goldsmith, W. S. .......... Atlanta.
Greenleaf, J. S. .......... Gifford, S. C.
Hardon, V. O. .......... Atlanta, Ga.
Hanger, F. M. .......... Staunton, Va.
Hobbs, A. G. .......... Atlanta.
Hoke, Michael .......... Atlanta.

Hubbard, T. V. . . . . . . . . . . . . . . . . . . . . . . . . . . . . . . . . . . . . . . . . . . . . Atlanta.
Hudson, W. H. . . . . . . . . . . . . . . . . . . . . . . . . . . . . . . . . . . . . . . . . LaFayette, Ala.
Hutchins, M. B. . . . . . . . . . . . . . . . . . . . . . . . . . . . . . . . . . . . . . . . . . . . Atlanta.
Jackson, E. B. . . . . . . . . . . . . . . . . . . . . . . . . . . . . . . . . . . . . . . . . Houston, Tex.
Johnston, Geo. Ben. . . . . . . . . . . . . . . . . . . . . . . . . . . . . . . . . . . . . Richmond, Va.
Johnson, J. Clarence. . . . . . . . . . . . . . . . . . . . . . . . . . . . . . . . . . . . . . . Atlanta.
Keister, B. C. . . . . . . . . . . . . . . . . . . . . . . . . . . . . . . . . . . . . South Boston, Va.
Kime, R. R. . . . . . . . . . . . . . . . . . . . . . . . . . . . . . . . . . . . . . . . . . . . . . Atlanta.
LeGrand, J. C. . . . . . . . . . . . . . . . . . . . . . . . . . . . . . . . . . . . . Birmingham, Ala.
Liell, E. N. . . . . . . . . . . . . . . . . . . . . . . . . . . . . . . . . . . . . . . Jacksonville, Fla.
Ludlow, Ogden C. . . . . . . . . . . . . . . . . . . . . . . . . . . . . . . . . . . . . . . . New York.
Manly, T. H. . . . . . . . . . . . . . . . . . . . . . . . . . . . . . . . . . . . . . . . . . . New York.
McRae, F. W. . . . . . . . . . . . . . . . . . . . . . . . . . . . . . . . . . . . . . . . . . . . Atlanta.
Minter, H. G . . . . . . . . . . . . . . . . . . . . . . . . . . . . . . . . . . . . . . . . . . . Jakin, Ga.
Mitchell, T. E. . . . . . . . . . . . . . . . . . . . . . . . . . . . . . . . . . . . . . . Columbus, Ga.
Moore, J. H. . . . . . . . . . . . . . . . . . . . . . . . . . . . . . . . . . . . . . . . Fair Play, S. C.
Mortimer, W. G. . . . . . . . . . . . . . . . . . . . . . . . . . . . . . . . . . . . . . . . New York.
Olmsted, J. C. . . . . . . . . . . . . . . . . . . . . . . . . . . . . . . . . . . . . . . . . . . . Atlanta.
Patterson, A. B. . . . . . . . . . . . . . . . . . . . . . . . . . . . . . . . . . . . . . . . . . . Atlanta.
Reeves, C. S. . . . . . . . . . . . . . . . . . . . . . . . . . . . . . . . . . . . . . . . . . . . . . Texas.
Robinson, G. P. . . . . . . . . . . . . . . . . . . . . . . . . . . . . . . . . . . . . . . . . . . Atlanta.
Roy, G. G. . . . . . . . . . . . . . . . . . . . . . . . . . . . . . . . . . . . . . . . . . . . . . Atlanta.
Roy, Dunbar. . . . . . . . . . . . . . . . . . . . . . . . . . . . . . . . . . . . . . . . . . . . . Atlanta.
Rumbold, Thos. F . . . . . . . . . . . . . . . . . . . . . . . . . . . . . . . . . . . . . St. Louis, Mo.
Smith, W. Monroe. . . . . . . . . . . . . . . . . . . . . . . . . . . . . . . . . . . . . . . . . Atlanta.
Stockard, C. C. . . . . . . . . . . . . . . . . . . . . . . . . . . . . . . . . . . . . . . . . . . Atlanta.
Toepel, Theo. . . . . . . . . . . . . . . . . . . . . . . . . . . . . . . . . . . . . . . . . . . . Atlanta.
Travis, W. D. . . . . . . . . . . . . . . . . . . . . . . . . . . . . . . . . . . . . . . . Covington, Ga.
Upshur, J. N. . . . . . . . . . . . . . . . . . . . . . . . . . . . . . . . . . . . . . . . Richmond, Va.
Valentine, Ferd C. . . . . . . . . . . . . . . . . . . . . . . . . . . . . . . . . . . . . . . New York
Vaughan, R. T. . . . . . . . . . . . . . . . . . . . . . . . . . . . . . . . . . . . Hot Springs, Ark.
Wiggin, F. H. . . . . . . . . . . . . . . . . . . . . . . . . . . . . . . . . . . . . . . . . New York.
Wood, B. J. . . . . . . . . . . . . . . . . . . . . . . . . . . . . . . . . . . . . . . . . . . . . Atlanta.
Woodward, J. F. . . . . . . . . . . . . . . . . . . . . . . . . . . . . . . . . . . . . . . Norfolk, Va.

